# Control of a Wheelchair-Mounted 6DOF Assistive Robot With Chin and Finger Joysticks

**DOI:** 10.3389/frobt.2022.885610

**Published:** 2022-07-22

**Authors:** Ivan Rulik, Md Samiul Haque Sunny, Javier Dario Sanjuan De Caro, Md Ishrak Islam Zarif, Brahim Brahmi, Sheikh Iqbal Ahamed, Katie Schultz, Inga Wang, Tony Leheng, Jason Peng Longxiang, Mohammad H. Rahman

**Affiliations:** ^1^ Department of Computer Sciences, University of Wisconsin-Milwaukee, Milwaukee, WI, United States; ^2^ Department of Mechanical Engineering, University of Wisconsin-Milwaukee, Milwaukee, WI, United States; ^3^ Department of Computer Sciences, Marquette, Milwaukee, WI, United States; ^4^ Electrical Engineering Department, Collège Ahuntsic, Montreal, QC, Canada; ^5^ Assistive Technology Program, Clement J. Zablocki VA Medical Center, Milwaukee, WI, United States; ^6^ Department of Rehabilitation Sciences & Technology, University of Wisconsin-Milwaukee, Milwaukee, WI, United States; ^7^ UFACTORY Technology Co., Ltd., Shenzhen, China

**Keywords:** assistive robot, 6DOF, multimodal control, wheelchair, motor dysfunction, activities of daily living

## Abstract

Throughout the last decade, many assistive robots for people with disabilities have been developed; however, researchers have not fully utilized these robotic technologies to entirely create independent living conditions for people with disabilities, particularly in relation to activities of daily living (ADLs). An assistive system can help satisfy the demands of regular ADLs for people with disabilities. With an increasing shortage of caregivers and a growing number of individuals with impairments and the elderly, assistive robots can help meet future healthcare demands. One of the critical aspects of designing these assistive devices is to improve functional independence while providing an excellent human–machine interface. People with limited upper limb function due to stroke, spinal cord injury, cerebral palsy, amyotrophic lateral sclerosis, and other conditions find the controls of assistive devices such as power wheelchairs difficult to use. Thus, the objective of this research was to design a multimodal control method for robotic self-assistance that could assist individuals with disabilities in performing self-care tasks on a daily basis. In this research, a control framework for two interchangeable operating modes with a finger joystick and a chin joystick is developed where joysticks seamlessly control a wheelchair and a wheelchair-mounted robotic arm. Custom circuitry was developed to complete the control architecture. A user study was conducted to test the robotic system. Ten healthy individuals agreed to perform three tasks using both (chin and finger) joysticks for a total of six tasks with 10 repetitions each. The control method has been tested rigorously, maneuvering the robot at different velocities and under varying payload (1–3.5 lb) conditions. The absolute position accuracy was experimentally found to be approximately 5 mm. The round-trip delay we observed between the commands while controlling the xArm was 4 ms. Tests performed showed that the proposed control system allowed individuals to perform some ADLs such as picking up and placing items with a completion time of less than 1 min for each task and 100% success.

## 1 Introduction

Impairments in arm function are common in individuals with neurological or orthopedic disorders and significantly impact health-related quality of life (Cowan et al., 2012; McKee and Daneshvar, 2015; Mlinac and Feng, 2016; Taylor, 2018; Alizadeh et al., 2019; Minetto et al., 2020). Potential causes are conditions such as stroke, cerebral vascular accident, amyotrophic lateral sclerosis, spinal cord injury (SCI), trauma, and occupational injuries (McKee and Daneshvar, 2015). These incidents may cause the affected person to lose upper limb functionality, which significantly limits functional independence and obstructs them from doing their activities of daily living (ADLs) without external help. Current estimates of need and unmet need for self-care personal assistance may indicate future demand for long-term care services.

There is a growing need for assistive robots and devices that support the independent life of elderly and disabled people. In recent years, wheelchair-mounted robotic arms and other physically assistive robotic devices have provided a promising solution to assist individuals with upper limb impairments who cannot perform simple daily tasks such as eating and drinking a cup of water independently (Tangcharoensathien et al., 2018; Toro-Hernández et al., 2019; Valk et al., 2019). However, to be effective, these assistive robots must be easily controllable by their users and have a user-friendly interface (Jiang et al., 2013; Rabhi et al., 2013; Rabhi et al., 2015; Craig et al., 2016; Penkert et al., 2021).

This manuscript described a chin joystick and finger joystick system in Cartesian mode to control a wheelchair and wheelchair-mounted 6DoF robotic arm. The novelty of this study lies in three folds. The inverse kinematics of the robotic arm is solved using the steepest descent (Ruder, 2016) method. Moreover, the workspace for end-effector movement is determined based on the essential ADLs. Furthermore, an integrated control framework is proposed, which consists of developed software and circuitry for seamless control of a wheelchair and an assistive robotic arm. The relevance of this study lies in how it will help individuals with restricted mobility to not only transport indoors and outdoors but also interact with the world through a robotic assistive arm. The primary focus of the study was to develop the control framework and evaluate the performance of the developed system which will enable patient studies in the future. As a first and essential step to evaluate the control of the robot using the joysticks to perform ADLs, this study recruited healthy participants.

The rest of the study is organized as follows: Section 2 represents the related works in controlling the robotic arm with multimodal user input. Section 3 discusses the preliminaries of a robotic arm and its parameters, wheelchair, finger joystick, and chin joystick. Section 4 is dedicated to the theoretical analysis of the robotic arm’s forwarding kinematics, dynamics, and inverse kinematics and considered workspace and control architecture. Section 5 discusses the control. Section 6 presents the experimental setup. Section 7 discusses the experiments, results, and discussion. Section 8 gives the conclusion.

## 2 Related Works

Researchers have previously investigated how to assist people with disabilities to manipulate robotic arms using control modalities. A physical joystick is widely accepted among those modalities to control assistive devices such as a powered wheelchair and robotic arm. It is commercially available, inexpensive, and simple in design and can operate the end-effector through directed selection (Malkin et al., 2011). It provides two-dimensional control for the *x* and *y* axes, the most significant limitation of commercially available joysticks. To overcome this limitation, researchers came up with different control approaches as well as different designs.

Intelligent control is proposed (Rabhi et al., 2013) for a joystick to drive a wheelchair focusing on the security of the user. A combination of a fuzzy controller and vector field histogram is used to handle the obstacles and random situations of the wheelchair environment. In (Rofer et al., 2009), the intelligent driving assistance algorithm controls a motorized wheelchair via a head-joystick, altering the translational and rotational velocities. It also prevents all collisions along the way.

In Fall et al. (2015), Thorp et al. (2015), Fall et al. (2018), Penaloza and Nishio (2018), Ansari et al. (2019), and Francis et al. (2021), body–machine interface (BMI) was developed, taking advantage of the user’s residual motion. They extract the user input from the kinematics of both neck and shoulder to then produce a proportionally controlled signal and drive the power wheelchair. A BMI shown in Baldi et al. (2017) was based on head tilt estimation and EMG signal. In Rudigkeit and Gebhard (2019), an adaptive head motion system allows the user to control both an assistive robotic arm as well as a visual interface in a computer. Another head gesture recognition method is implemented by Solea et al. (2019) using a head pose control algorithm to operate a power wheelchair. The control system translates the head motions into speed and directional device control. Using the alternative input port of the power wheelchair, nonmechanical solutions can be used based on tracking the user’s head movements. An egocentric camera is used as the primary sensor, and computer vision technology is used to track the user’s head motion and translate it to control signals for the wheelchair (Kutbi et al., 2017). Users with disabilities can also drive electric-powered wheelchairs with force-sensing joysticks operated via the chin. Compared to a conventional joystick, they do not need to move their heads quickly and accurately (Guo et al., 2002). Sometimes users feel uncomfortable/awkward after using a chin joystick control. People with impaired upper limb function who currently use switch-based head controls or chin joysticks may find that the intelligent glass device could be an alternative (Penkert et al., 2021).

To operate a robotic arm as an assistive device, a 3D joystick was developed (Jiang et al., 2013). An optimized joystick control interface is proposed by Rabhi et al. (2015) using a neural network. Another control approach is gesture control, which uses hand motion concerning the wrist for different commands. For gesture recognition, it used Kinect sensors (Ren et al., 2013), vision sensors (Zhou et al., 2013), and ultrasonic sensors (Kalgaonkar and Raj, 2009). Laser-based sensors for recognition are used by Perrin et al. (2004). Try et al. (2021) used visual sensor fusion to perform assistive drinking. Different algorithms, as well as techniques such as fuzzy logic (Pulikottil et al., 2018), neural network (Alonso-Martín and Salichs, 2011), hidden Markov model (Memon et al., 2016), pattern matching (Nishimori et al., 2007), histograms (Andreasen Struijk et al., 2017), and graph matching (Hildebrand et al., 2019), are used in this approach. To control the assistive devices and to assist physically disabled persons, a voice-controlled-based powered wheelchair system is also developed (Nishimori et al., 2007; Alonso-Martín and Salichs, 2011; Memon et al., 2016; Pulikottil et al., 2018). The user can control the wheelchair by voice commands. Other alternative control methods such as the tongue (Andreasen Struijk et al., 2017; Hildebrand et al., 2019), eye tracking (Păsărică et al., 2015), and the combination were used. By using brain-controlled wheelchairs, patients could take control of their wheelchairs by their thoughts, thus regaining mobility function. This system will allow users to drive an electric wheelchair using a brain–computer interaction system interfaced with a navigation system (Puanhvuan et al., 2017).

Incorporating both the advantages and disadvantages of the above-mentioned solutions, this work proposes an approach without adding additional sensors and processing power, which will increase cost and complexity. In order to understand and integrate the proposed control framework with an assistive arm and a wheelchair, minimal additional hardware is required, and the learning curve is kept shorter for new users and existing users of a system equivalent to the proposed solution.

## 3 Preliminaries

### 3.1 Robotic Arm

**FIGURE 1:**
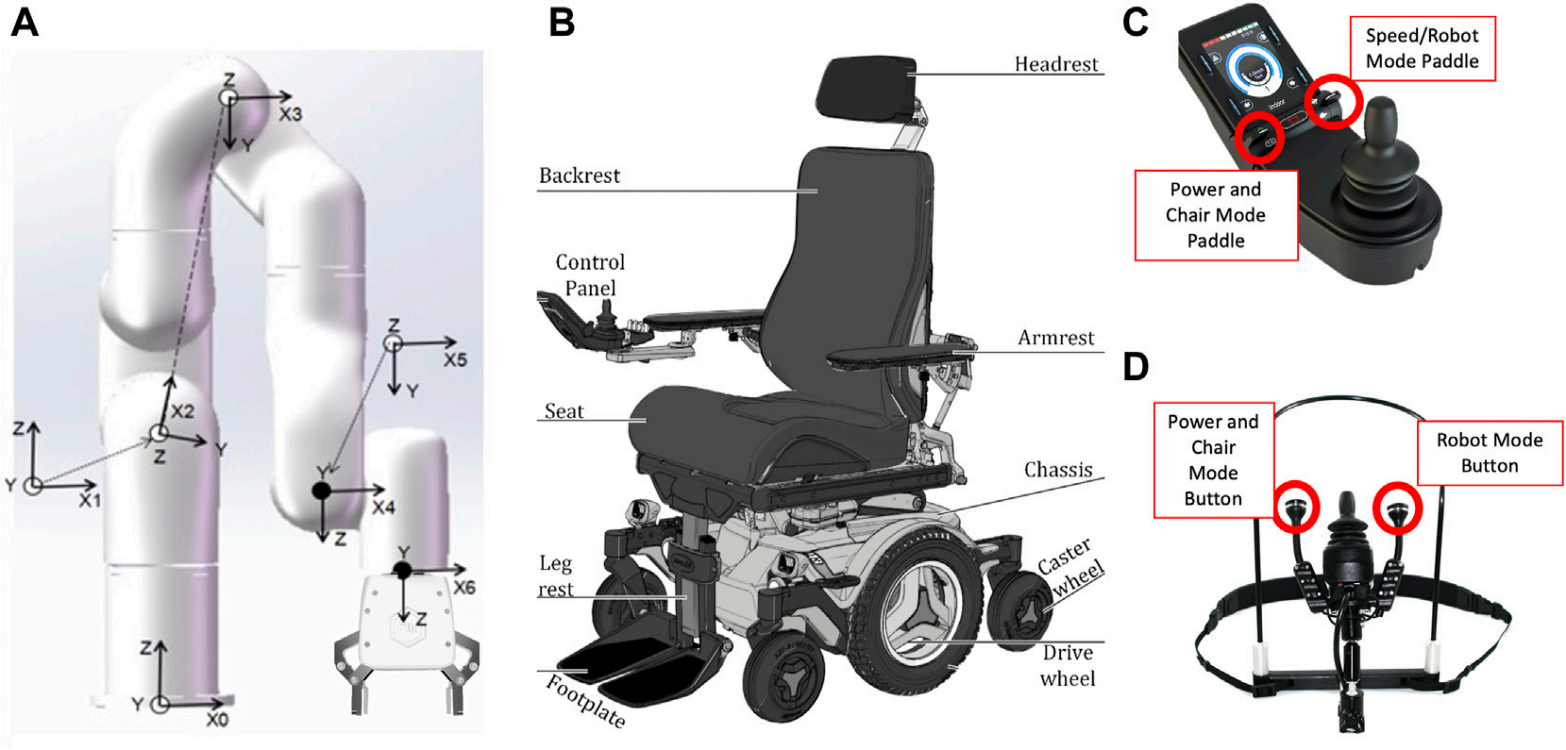
**(A)** Joint coordinate definition (36, 2022). **(B)** Overview of Permobil M3 corpus. **(C)** Finger joystick. **(D)** Chin joystick.

The xArm 6 robotic manipulator from UFactory was mounted on a power wheelchair (xArm Collaborative Robot, 2022) to give ADL help during the research. This versatile robotic arm is composed of six brushless motors with harmonic reducers that work together to manipulate objects of up to 5 Kg with an absolute position accuracy of 5 mm. It has RS-485 (Lizhong et al., 2010) communication mode and Modbus RTU protocol (Peng et al., 2008). It brings to the user multiple closed-loop control methods including current, torque, speed, and position, which could be tuned to achieve faster, smoother, and more precise movements. The coordinate axes of the arm can be seen in Figure 1A, where each axis subnumber represents each one of the six actuators contained in the arm.

### 3.2 Power Wheelchair

A Permobil M3 Corpus power wheelchair was used to carry out the experiments in this research (Permobil, 2022c) (see Figure 1B). This powered wheelchair shows a finely tuned product that not only allows its users to smoothly transport from point A to B with an intuitive response to the user input but it also brings the commodity of adjusting the seat height and tilt to support the needs of its possible users. Thanks to the R-net system used in the wheelchair, multiple modules can be added to expand functionality; for this case, an input/output module was used to extract the user commands from the joysticks into the control computer.

### 3.3 Finger Joystick

The finger joystick used in this research is shown in Figure 1C. The joystick used in this control system, developed by Curtiss-Wright and sold by Permobil (Permobil, 2022b), has advantages by offering a two degree of freedom joystick and two control paddles, one on the left to power the system and switch the chair operation modes while the right paddle changes the wheelchair movement speed. It also has four programable buttons. The system has a program that changes between power wheelchair control and assistive arm manipulation through fixed buttons. When the system enters the assistive arm mode, the signals from the joystick will change the arm’s position only based on presets of dimensions of movement (X-Y or Y-Z planes, for example). Using the speed paddle, the user can switch between the six possible modes (X-Y axes, Z-Yaw axes, roll-pitch axes, and gripper mode) to manipulate the robot arm and the gripper attached to the end of the arm.

### 3.4 Chin Controller Joystick

The chin joystick used in this research is shown in Figure 1D. Compact chin joysticks (Permobil, 2022a) are used for chin control, with standard proportional force and thrown in a small package. They are also equipped with remote ON/OFF and mode switches. This chin joystick brings the same base functionality as the finger joystick but is adjusted for patients with physical limitations in their hands and arm. It has two degrees of freedom to address the user’s desire to manipulate the power wheelchair and the assistive robotic arm. At the same time, the switches attached to its ports allow power ON/OFF the system and move between wheelchair and active arm control.

## 4 Theoretical Analysis

### 4.1 Kinematics of xArm 6 Robot

This section deals with the computation of the forward kinematics (Sunny et al., 2021), by taking the joint angles it returns the end-effector position of the arm. Modified Denavit–Hartenberg (DH) parameters were used to solve the direct kinematics and the results can be seen in Table1.

**TABLE 1:** Modified Denavit–Hartenberg parameters for xArm 6.

*i*	*a*_*i*_	*α*_*i*_	*d*_*i*_	*θ*_*i*_
1	0	0	*L*_1_	*θ*_1_
2	0	− *π*/2	0	θ2+θ20
3	*L*_2_	0	0	θ3+θ30
4	*L*_3_	− *π*/2	*L*_4_	*θ*_4_
5	0	*π*/2	0	*θ*_5_
6	*L*_5_	− *π*/2	*L*_6_	*θ*_6_

It needs to be considered that *L*_*i*_ is the distance between the current joint (*i*) and the previous one and θi0 is the angular offset of the *i*th joint with respect to the previous one in the X-axis. Table 2 has values that describe the robotic arm in modified DH parameters.

**TABLE 2:** Dimensional parameters of xArm 6.

*L*_1_	*L*_2_	*L*_3_	*L*_4_	*L*_5_	*L*_6_	θ20	θ30
267	289.49	77.5	342.5	76	97	1.3849	1.3849
mm	mm	mm	mm	mm	mm	Rad	rad

Note that the terms *a*_*i*_ (distance from the X-axis of the frame i to the X-axis of the frame i-1), *α*_*i*_ (the angle between the Z-axis of the frame i and the Z-axis of the frame i-1), *d*_*i*_ (distance from the Z-axis of the frame i to the Z-axis of the frame i-1), and *θ*_*i*_ (the angle between the X-axis of the frame i and the X-axis of the frame i-1) follow the modified DH convention as presented in (McKerrow, 1993). We get the transformation matrix as follows:

Tii−1=cosθi−sinθi0αicosαi⁡sinθicosαi⁡cosθi−sinαi−d⁡sinαisinαi⁡sinθisinαi⁡cosθicosαidi⁡cosαi0001 (1)

In Eq. 1, Tii−1 is the transformation matrix that the closest frames/joints in the robotic arm have in common to represent position and orientation in the perspective of the previous joint. This transformation matrix can and is concatenated to determine the end-effector position and orientation based on all the frames/joints that came before it, as seen in Eq. 2.

T60=T10T21T32T43T54T65 (2)

**FIGURE 2:**
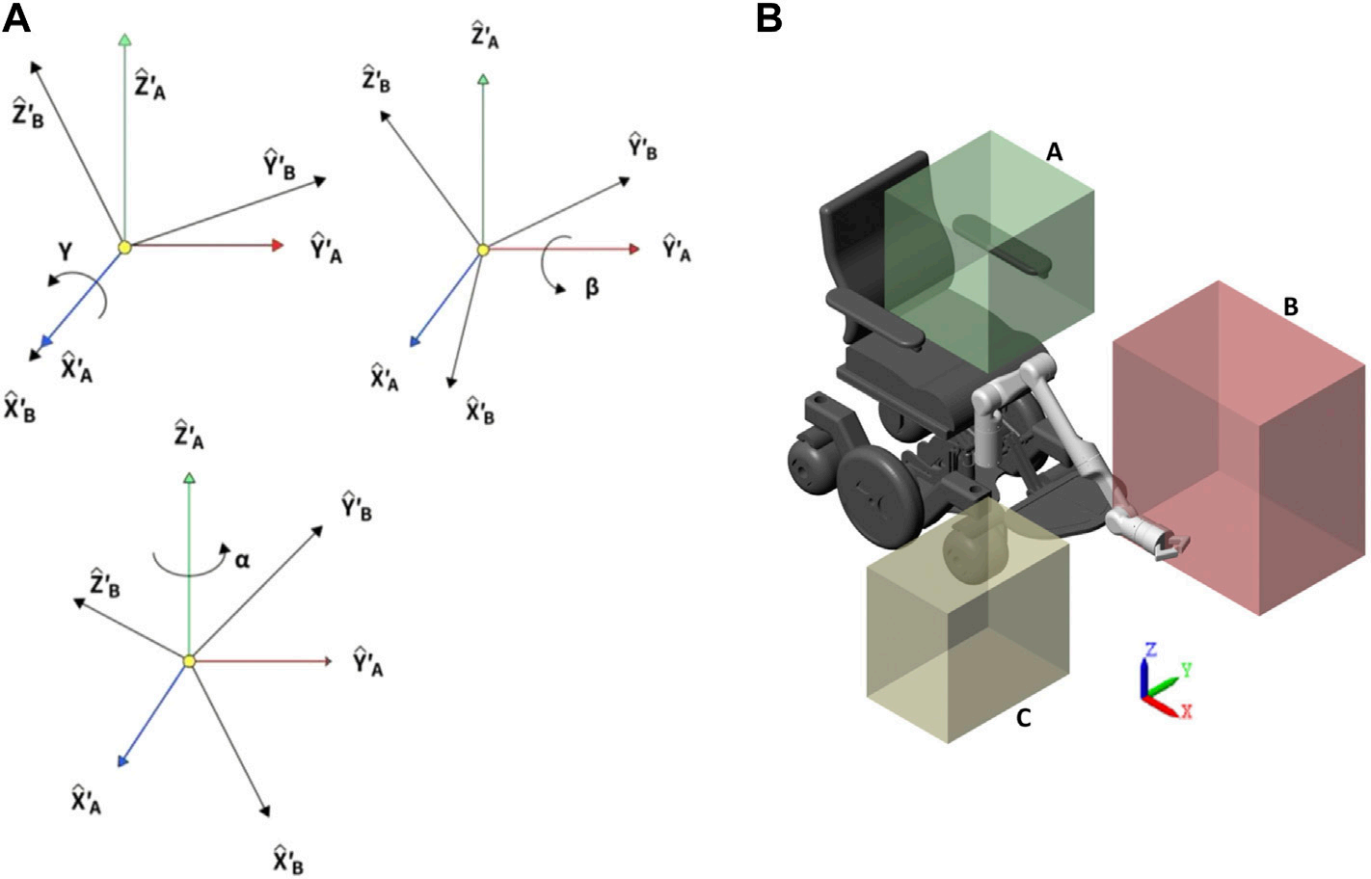
**(A)** Roll, pitch, and yaw angle. **(B)** Considered workspace for daily living activities.

To understand the equal rotation matrix 3 that shows the orientation of a plane {*B*} relative to a plane {*A*}, a breakdown of its components is needed. In Figure 2A, the roll angle (*γ*) changes along the X-axis, the pitch angle (*β*) changes along the Y-axis, and the yaw angle (*α*) changes along the Z-axis. Knowing this, a product of the rotation in the three axes as shown in Eq. 3 produces the same rotation matrix as that when multiplied by a position vector in plane {*B*} returns the position vector as seen in plane {*A*}; this happens because the angles of rotation (*γ*, *β* and *α*) go along the axes of the plane {*A*}.

RXYZBAγ,β,α=RZαRYβRXγ (3)

### 4.2 Inverse Kinematics

Inverse kinematics is the computation of the joint positions given the pose of the end-effector by solving a nonlinear set of transcendental equations. Thus, the inverse kinematics of the xArm 6 robotic arm was found using the steepest descent method (Ruder, 2016). For this purpose, let the pose of the robot be obtained from Eq. 2 as f(θ)=pose(T60) , where:

$$\theta =\left[\begin{array}{cccccc} \theta_{1} & \theta_{2} & \theta_{3} & \theta_{4} & \theta_{5} & \theta_{6} \end{array}\right]$$ (4)

Then, given a desired goal pose $g\in R^{6}$ :

$$g=\left[\begin{array}{cccccc} \theta_{x} & \theta_{y} & \theta_{z} & x & y & z \end{array}\right]$$ (5)

where *x*, *y*, and *z* are the end-effector position relative to coordinate frame {0} and *θ*_*x*_, *θ*_*y*_ and *θ*_*z*_ are the orientation of the end-effector using Euler angles. Then, we seek to find the joint angles *θ* ^∗^ that minimizes the difference between the goal pose and the end-effector pose:

$$\begin{array}{ll} \theta^{*} & =\underset{\theta }{\min}\frac{1}{2}\|g-f\left(\theta \right)\|\\ & =\underset{\theta }{\min}G\left(\theta \right) \end{array}$$ (6)

Eq. 6 is the objective function, the solution to this minimization problem yields the inverse kinematics of the xArm 6. The inverse kinematics solution is then obtained by applying the steepest descent pseudo-code found in Algorithm 1. The parameters used are adaptive step size, 1 mm; tolerance, 0.1 mm; and convergence delay, 0.2 s.

Algorithm 1 Steepest descent method.

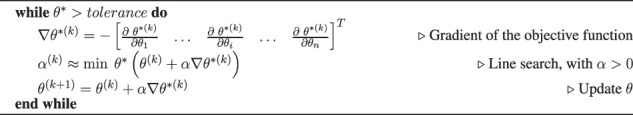

### 4.3 Workspace Consideration

For the ADL, some considerations regarding the workspace were taken as Figure 2B shows three subworkspaces. The split in the workspace was made to guarantee a proper operation of the robotic arm in the multiple ADLs to be tested. Workspace A is the closest to the individual which implies tight restrictions along the *X* and *Z* axes. The workspace B is the furthest away from the wheelchair and is mainly intended to grab objects of middle and higher levels like tables or shelves on the positive X-axis. In conclusion, workspace C covers an area closer to the ground, and thus, it gives more priority to movements along the global Z-axis.

### 4.4 Control Architecture

**FIGURE 3:**
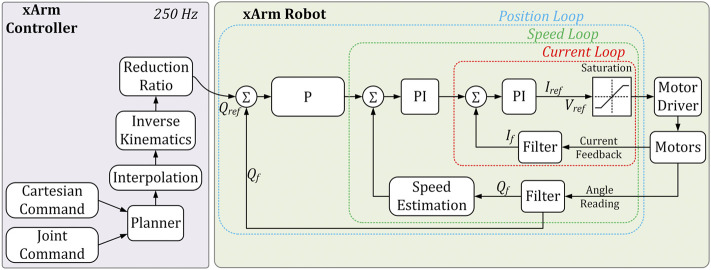
Control architecture of the system.

The control architecture (Sunny et al., 2021) for the robotic arm can be seen in Figure 3. The computation platform used is an Intel Celeron Dual Core Processor J1900 (xArm Control box) with a real-time Linux operating system. A control loop in the system will imply taking the reference joint/Cartesian positions given by the user along with a path planning layer, inverse kinematics layer, and reduction ratio layer that translates into reference position, *Q*_*ref*_, for all the motors and thus enter into the position controller which has a proportional (P) controller. As seen in the control architecture, the speed is estimated (derived from the second-order filtering) from the filtered position data (*Q*_*f*_). Within the motor drivers, the position loop decomposes the signal into velocities through a proportional–integral (PI) controller, and those are then translated into currents *I*_*ref*_ through another PI controller that will send them as logical voltage *V*_*ref*_ to operate the motors. The current sensor (Allegro MicroSystems) and encoder (absolute magnetic 16-bit multiturn) are used to measure the motor current and the robot joint angle, respectively. As seen in the schematic in Figure 3, the current loop runs at 0.1 ms, whereas the over control loop runs at 250 Hz or 4 ms.

To reduce the noise in the signals obtained by the current and encoder sensors a second-order filter was used in the motor drivers. In summary, the control architecture implements a three-layered approach that compares the position, velocity, and current of the motors to guarantee a low error and fast response to the user input.

## 5 Control

**FIGURE 4:**
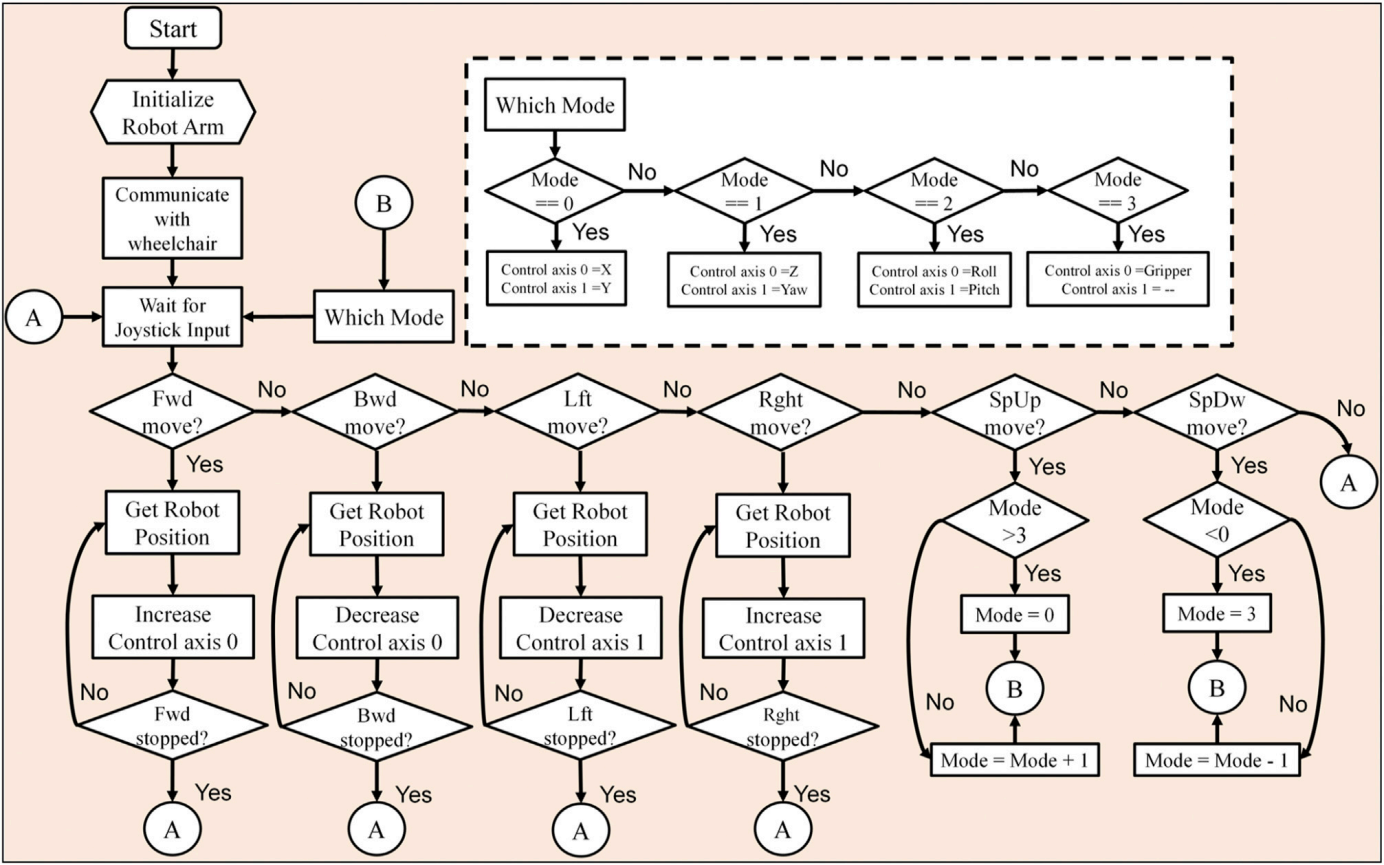
A flowchart summarizing the program to manipulate the xArm’s end-effector position.

Figure 4 shows the flowchart outlining the program to manipulate the assistive robotic arm’s end-effector position. Once the user starts the robotic system, it will preload the first operation mode, where the assistive arm can be moved on the X–Y axes. Movements along the X–Y axes are forward (Fwd), backward (Bwd), left (Lft), and right (Rght), which can be achieved by pressing the chin joystick’s head switches. The user will change the operation modes to access the three-dimensional displacement changing from X–Y to Z direction (up/down), the three rotational movements (roll, pitch, and yaw), and the control over the gripper. In order to change the operation modes, the user needs to either move the speed paddle up (SpUp) or down (SpDwn).

## 6 Experimental Setup

**FIGURE 5:**
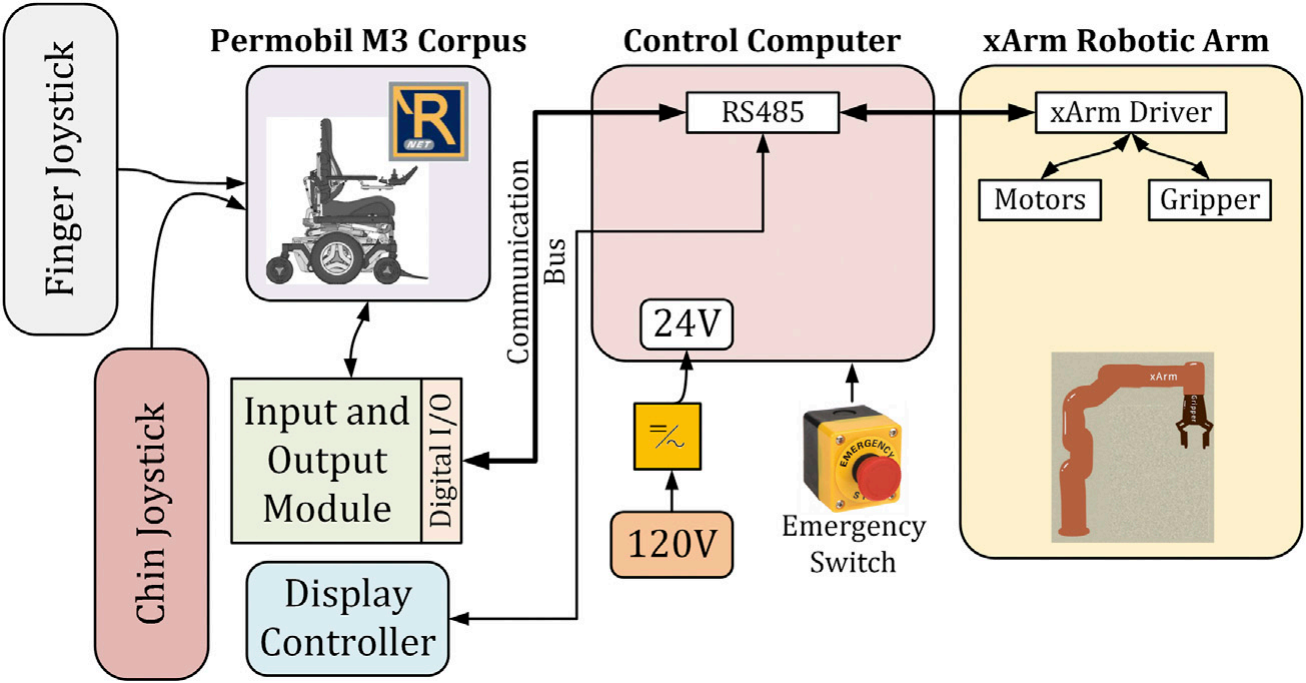
Block diagram of the experimental setup.

Figure 5 presents the diagram of the experimental setup of a power wheelchair with an assistive robotic arm attached to it. The first section contains the user input devices such as the finger joystick and chin joystick from left to right. The next section contains the power wheelchair Permobil M3 Corpus which runs the R-net system (Wright, 2022) for the transportation control and status display. Over the input/output module (Al-Wakeel and Ilyas, 1992), the R-net system sends and receives control signals from the control computer. The main section is the control computer where multiple Python programs run to coordinate the control of the power wheelchair and assistive robotic arm as well, a defining characteristic of this layer is the digital and analog input/output connectors that physically connect this section with the rest. In conclusion, there is a robotic arm section with the xArm 6, which takes both power and control signals from the computer section and returns the current status of the arm. This layer is composed of the gripper and the brushless DC motors with reduction boxes and motor drivers over a Modbus communication network; the three main loops shown on the right side of Figure 3 are contained here.

The setup is an interconnected system with custom circuitry and software that seamlessly lets the user manipulate a wheelchair and an assistive robot arm. The Python program uses a dedicated Software Development Kit to control the robot arm based on the user signals of the finger joystick and chin joystick giving individuals with restricted mobility the possibility of performing some ADL.

## 7 Experiments, Results, and Discussion

This research presents pick and place tasks from three different locations: ground, table, and shelf, for which 10 participants took 10 repetitions. All the data from the participants were retained for analysis as none of these samples involved invalid responses or timeouts. Two studies were approved by the UWM IRB (21.306. UWM and 21.309. UWM) to determine the performance of the proposed finger joystick and chin joystick system. Ten healthy participants were recruited to maneuver a wheelchair-mounted xArm 6 robot using a finger joystick and a chin joystick for this study. Table 3 shows the details of the participants of this study.

**TABLE 3:** Characteristics of the 10 healthy participants.

Characteristics	Value
Age (years)	
Mean	27.8
Standard Deviation	2.9
Gender	
Male	10
Female	0

**FIGURE 6:**
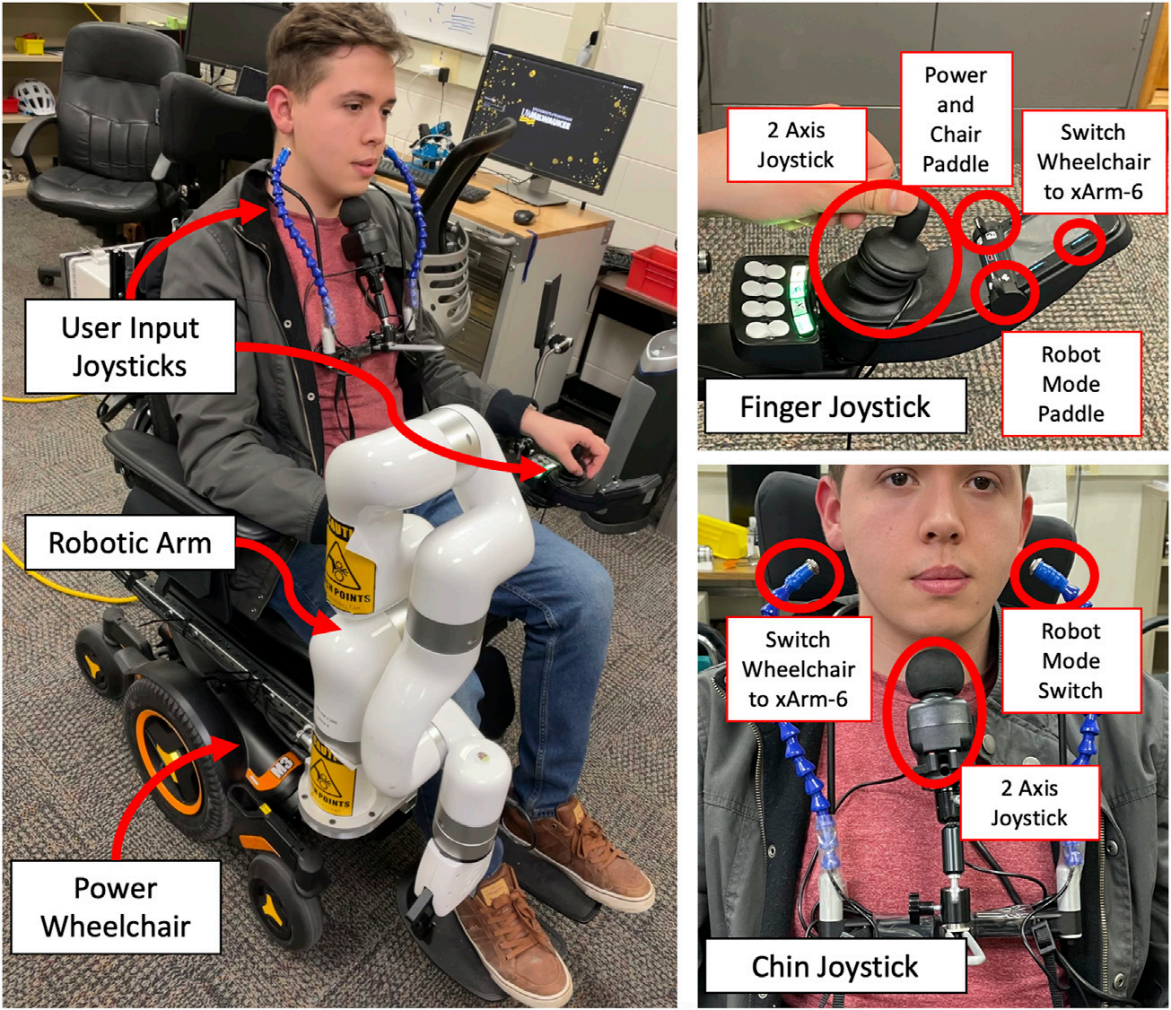
Participants use the system while seated in the wheelchair.

Figure 6 shows the final setup of the system. The power wheelchair with the assistive robotic arm is mounted on the right side rail, and one participant can be seen from left to right. Two user inputs control the robotic arm and the powered wheelchair. Finger joystick control is done with a two-axis joystick. There is a toggle switch to change the control between the wheelchair and the xArm 6.

**FIGURE 7:**
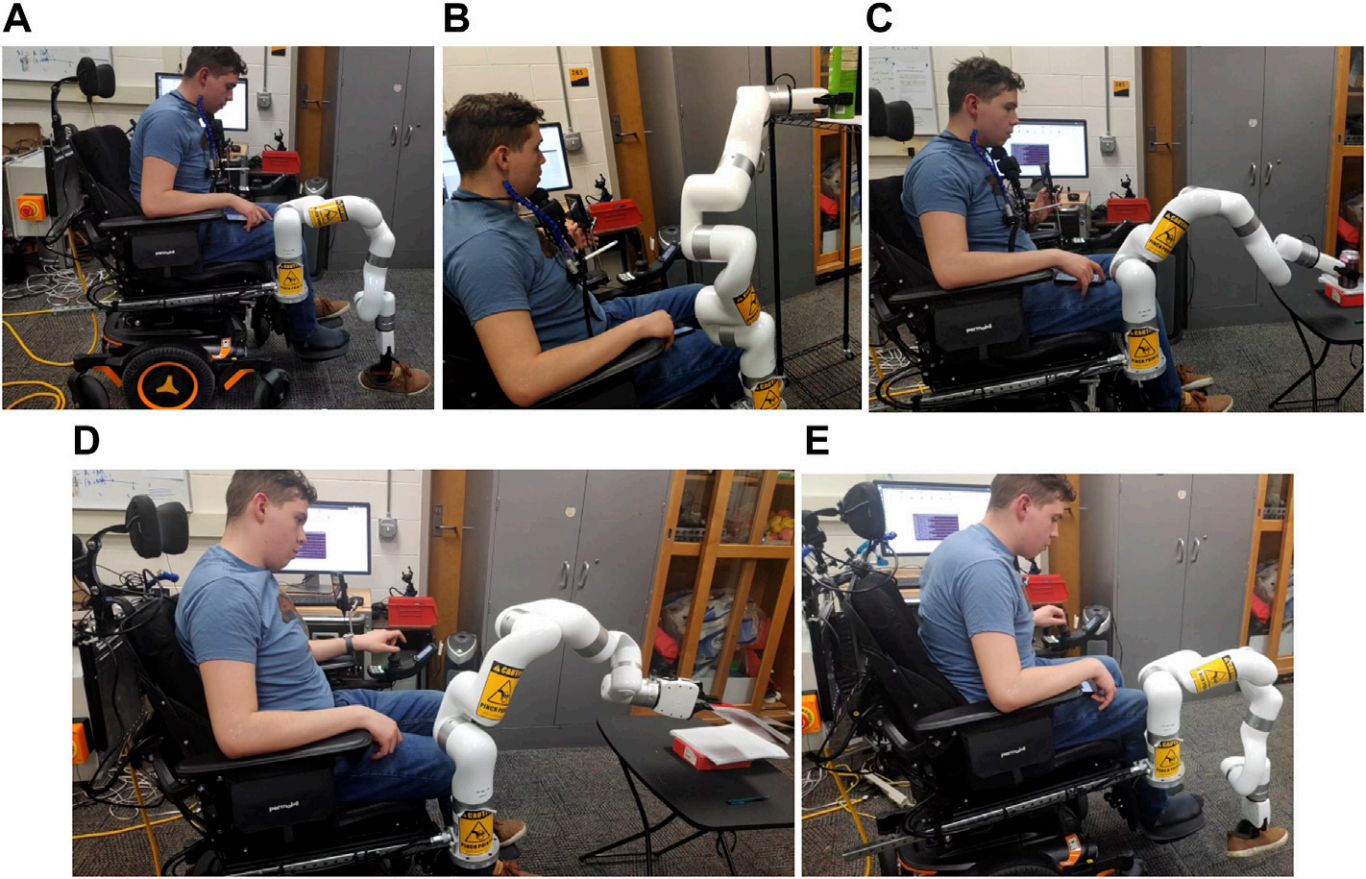
ADL experiments picking objects from the ground **(A,E)**, the shelf **(B)**, and the table **(C,D)**.

**FIGURE 8:**
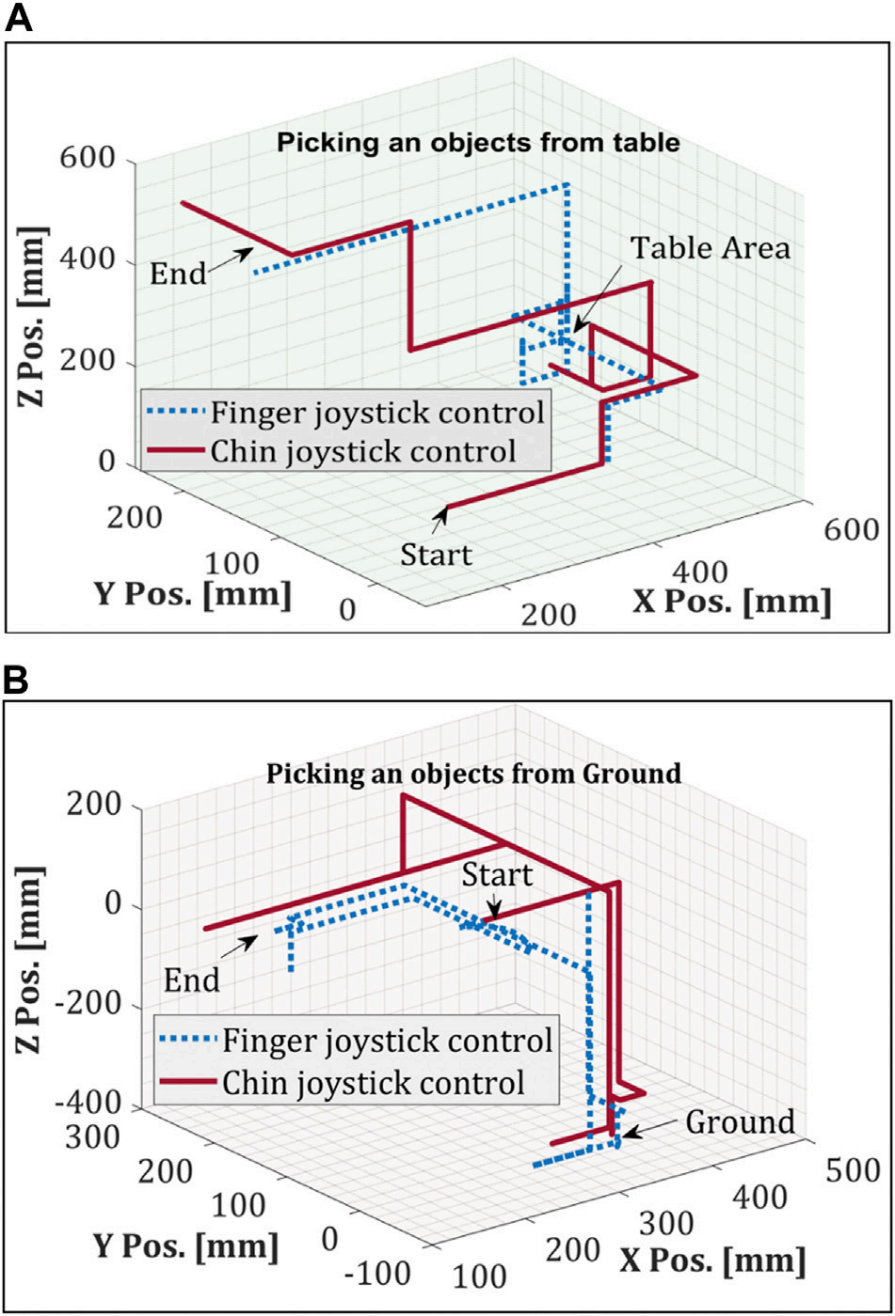
Cartesian trajectory of picking an item from **(A)** table and **(B)** ground.

Likewise, for chin joystick control, a two-axis joystick operated by chin movement and the head array switches are configured to change the mode between the wheelchair and robotic arm. First, participants were trained for 10 min with a finger joystick and chin joystick. For safety measures, the robot arm’s active workspace is reduced to avoid any contact with the user. As daily living tasks, picking and placing items from the ground, a table and shelf were selected due to their change in height. The data from the robotic arm was gathered for both finger joystick and chin joystick experiments allowing to display of the trajectory of the robot gripper. Figures 7A–E show a participant performing the proposed ADL tasks where the highest item was 1.5 m above the ground, and the furthest away object horizontally was 0.61 m. In these tasks, the Cartesian mode allows manipulating the robotic arm based on the theory seen in Section 4 by updating the target position using the joysticks. Figure 8A shows the trajectory followed by the robot gripper for both finger joystick and chin joystick to pick an object from the table. The solid line is the trajectory using the chin joystick, and the dotted line represents the path using a finger joystick. Likewise, Figure 8B shows the end-effector path to picking an object from the ground.

**FIGURE 9:**
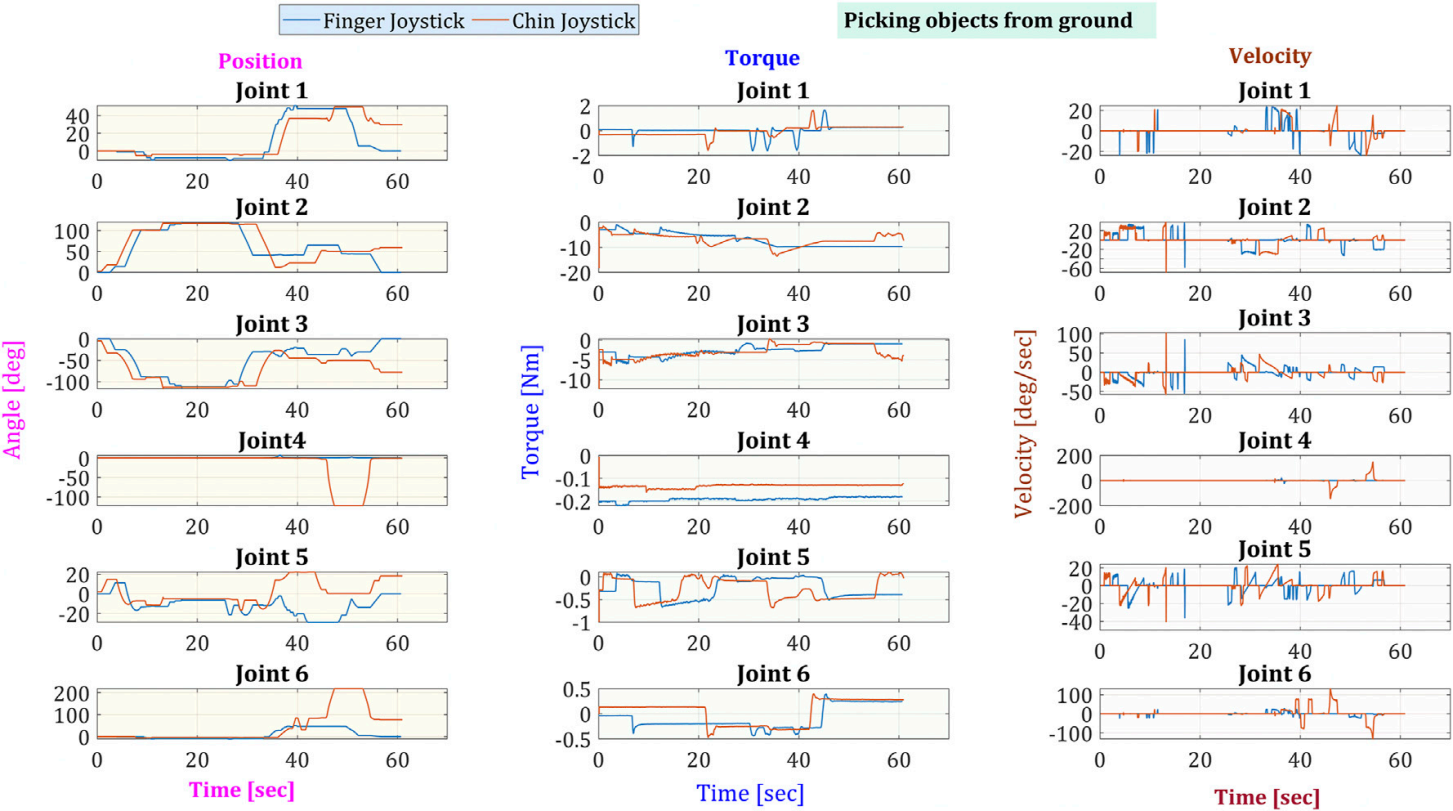
Angular position, torque, and speed of the six joints in the robotic arm while picking an object from the ground.

**FIGURE 10:**
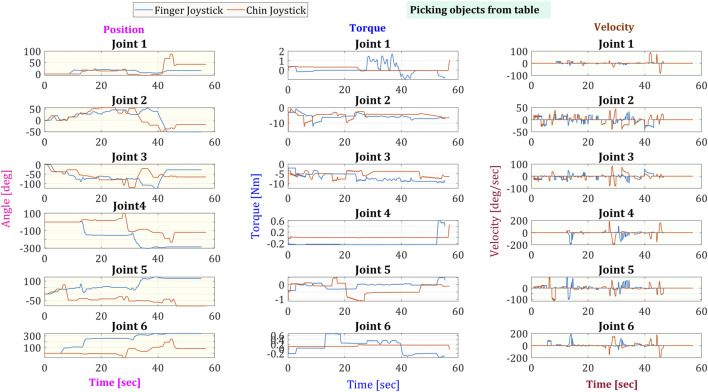
Angular position, torque, and speed of the six joints in the robotic arm while picking an object from the table.

From the gathered data of the robotic arm, the joint angles, torque/current consumption, and speed were extracted and shown in Figure 9 for picking from the ground and Figure 10 for picking an object from the table. Both figures can have similarities, like all joints share the same initial position. Joints 2 and 3 show the highest amounts of torque due to having to handle most of the weight of the arm. In conclusion, the last four joints share a higher speed range since they do not handle a great weight on the task, and thus, their torque stays lower.

**FIGURE 11:**
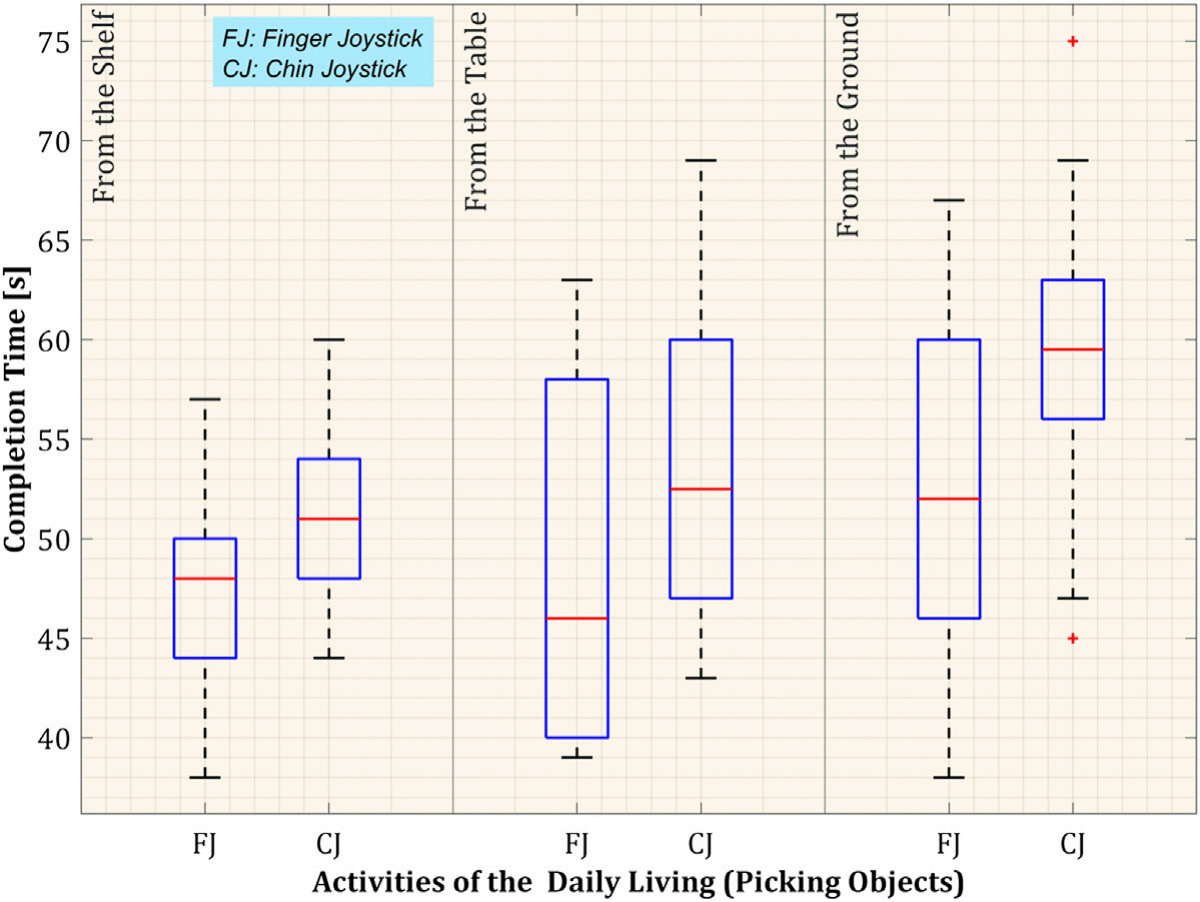
Distribution of task completion time.

Figure 11 presents the box plot of the distribution of task completion. The task of picking an object from a high shelf using the finger joystick had a minimum time of 38 s, whereas the maximum time was 57 s, and the median of the task was 48 s. However, with the chin joystick, the completion time was maximum = 60 s, minimum = 44 s, and median = 51 s. The comparison of time completion with both controls is presented in Table 4. Feedback was received from the participants at the end of the experiments through an experience evaluation form about the system’s comfortability, safety, usability, and functionality. The response was positive as they performed each task with ease improving in each iteration. Although participants provided positive feedback for the user-friendly control framework design, it cannot be generalized based only on healthy participants’ opinions. Feedback from individuals with restricted mobility is necessary to get a more solid conclusion about the proposed system in future studies. Stroke survivors and individuals with multiple sclerosis who have manual muscle testing score ≥4 , individuals with SCI levels: cervical nerves (C4—C8) and thoracic nerves (T1—T12) will be able to operate the system with the finger joystick and chin joystick. The steepest descent method works very well for this specific application, controlling an assistive robot with a chin joystick and a finger joystick because the proposed application requires slow to moderate robot movement to perform ADLs rather than fast movement as in industrial applications.

**TABLE 4:** Distribution of completion time of activities of daily living with both controls (N = 10).

Task	Completion time		
	Min (s)	Max (s)	Median (s)
**Picking** Object from the shelf			
Chin joystick	44	60	51
Finger joystick	38	57	48
**Picking** Object from the table			
Chin joystick	43	69	52.50
Finger joystick	39	63	46
**Picking** Object from the ground			
Chin joystick	47	69	59.50
Finger joystick	38	67	52

## 8 Conclusion

This study aimed to develop a multimodal control system to assist individuals with restricted mobility in performing ADL while having control over their mobility and environment. The workspace of a wheelchair and a wheelchair-mounted robotic arm are considered, combined, and then translated the control commands to control manually in Cartesian mode to accomplish this task. The proposed control system was put to the test with the participation of 10 healthy individuals. The steepest descent method proved to be useful in finding the inverse kinematics model of the robotic arm. For safety measures, the assistive robot arm workspace was limited to avoid contact with the user. Experiments results guarantee that the proposed control framework can suitably be used to maneuver both a powered wheelchair and an assistive robot for ADL assistance using the integrated wheelchair joysticks. This research thus contributes to significant technological advancement that has yielded a novel control framework with two different control modalities, including chin joystick and finger joystick. Note that the developed control framework and its validation with healthy participants demonstrating a hundred percent successful completion of ADL tasks with two different control modalities are the perquisites results before testing the system with individuals with upper extremity dysfunctions. The comparison between the finger joystick and chin joystick operations in ADLs is also demonstrated. Although the participants gave positive feedback for both modes of operation, the distribution of the task completion time indicates that the finger joystick operation was less time-consuming than the chin joystick control. The following steps for this research include expanding the user input devices and reducing task completion time. Moreover, future works will conduct experiments with wheelchair users having limited or no upper limb movements.

## Data Availability

The raw data supporting the conclusion of this article will be made available by the authors, without undue reservation.

## References

[B1] Al-Wakeel S. S. Ilyas M. (1992). R-Net: A High Speed Fibre Optics Network with Reservation Access Protocol. Int. J. Digit. Analog. Commun. Syst. 5, 1–13. 10.1002/dac.4510050102

[B2] Alizadeh A. Dyck S. M. Karimi-Abdolrezaee S. (2019). Traumatic Spinal Cord Injury: an Overview of Pathophysiology, Models and Acute Injury Mechanisms. Front. Neurol. 10, 282. 10.3389/fneur.2019.00282 30967837 PMC6439316

[B3] Alonso-Martín F. Salichs M. A. (2011). Integration of a Voice Recognition System in a Social Robot. Cybern. Syst. 42, 215–245. 10.1080/01969722.2011.583593

[B4] Andreasen Struijk L. N. S. Egsgaard L. L. Lontis R. Gaihede M. Bentsen B. (2017). Wireless Intraoral Tongue Control of an Assistive Robotic Arm for Individuals with Tetraplegia. J. Neuroeng Rehabil. 14, 110–118. 10.1186/s12984-017-0330-2 29110736 PMC5674819

[B5] Ansari M. F. Edla D. R. Dodia S. Kuppili V. (2019). Brain-computer Interface for Wheelchair Control Operations: An Approach Based on Fast Fourier Transform and On-Line Sequential Extreme Learning Machine. Clin. Epidemiol. Glob. Health 7, 274–278. 10.1016/j.cegh.2018.10.007

[B6] Baldi T. L. Spagnoletti G. Dragusanu M. Prattichizzo D. (2017). Design of a Wearable Interface for Lightweight Robotic Arm for People with Mobility Impairments. IEEE Int. Conf. Rehabil. Robot. 2017, 1567–1573. 10.1109/ICORR.2017.8009471 28814043

[B7] Cowan R. E. Fregly B. J. Boninger M. L. Chan L. Rodgers M. M. Reinkensmeyer D. J. (2012). Recent Trends in Assistive Technology for Mobility. J. Neuroeng Rehabil. 9, 20–28. 10.1186/1743-0003-9-20 22520500 PMC3474161

[B8] Craig T. L. Nelson C. A. Li S. Zhang X. (2016). “Human Gaze Commands Classification: A Shape Based Approach to Interfacing with Robots,” in 2016 12th IEEE/ASME International Conference on Mechatronic and Embedded Systems and Applications (MESA) (Auckland, NZ: IEEE), 1–6. 10.1109/mesa.2016.7587154

[B9] Fall C. L. Turgeon P. Campeau-Lecours A. Maheu V. Boukadoum M. Roy S. (2015). Intuitive Wireless Control of a Robotic Arm for People Living with an Upper Body Disability. Annu. Int. Conf. IEEE Eng. Med. Biol. Soc. 2015, 4399–4402. 10.1109/EMBC.2015.7319370 26737270

[B10] Fall C. L. Quevillon F. Blouin M. Latour S. Campeau-Lecours A. Gosselin C. (2018). A Multimodal Adaptive Wireless Control Interface for People with Upper-Body Disabilities. IEEE Trans. Biomed. Circuits Syst. 12, 564–575. 10.1109/tbcas.2018.2810256 29877820

[B11] Francis W. C. Umayal C. Kanimozhi G. (2021). Brain-computer Interfacing for Wheelchair Control by Detecting Voluntary Eye Blinks. Indonesian J. Electr. Eng. Inf. (IJEEI) 9, 521–537. 10.52549/ijeei.v9i2.2749

[B12] Guo S. Cooper R. Boninger M. Kwarciak A. Ammer B. (2002). “Development of Power Wheelchair Chin-Operated Force-Sensing Joystick,” in Proceedings of the Second Joint 24th Annual Conference and the Annual Fall Meeting of the Biomedical Engineering Society][Engineering in Medicine and Biology (Houston, TX, USA: IEEE), 2373–2374. vol. 3.

[B13] Hairong Jiang H. Wachs J. P. Pendergast M. Duerstock B. S. (2013). 3d Joystick for Robotic Arm Control by Individuals with High Level Spinal Cord Injuries. IEEE Int. Conf. Rehabil. Robot. 2013, 6650432. 10.1109/icorr.2013.6650432 24187250

[B14] Hildebrand M. Bonde F. Kobborg R. V. N. Andersen C. Norman A. F. Thogersen M. (2019). Semi-autonomous Tongue Control of an Assistive Robotic Arm for Individuals with Quadriplegia. IEEE Int. Conf. Rehabil. Robot. 2019, 157–162. 10.1109/ICORR.2019.8779457 31374623

[B15] Kalgaonkar K. Raj B. (2009). “One-handed Gesture Recognition Using Ultrasonic Doppler Sonar,” in 2009 IEEE International Conference on Acoustics, Speech and Signal Processing (Taipei, Taiwan: IEEE), 1889–1892. 10.1109/icassp.2009.4959977

[B16] Kutbi M. Chang Y. Mordohai P. (2017). “Hands-free Wheelchair Navigation Based on Egocentric Computer Vision: A Usability Study,” in Conference: Workshop on Assistance and Service Robotics in a Human Environment At: Vancouver, British Columbia, Canada.

[B17] Lizhong G. Peng W. Cheng M. Huibo J. (2010). Design and Implement of Rs485 High Speed Data Communications Protocol. Beijing, China: Tsinghua University Press.

[B18] Malkin J. Li X. Harada S. Landay J. Bilmes J. (2011). The Vocal Joystick Engine v1.0. Comput. Speech & Lang. 25, 535–555. 10.1016/j.csl.2010.03.005

[B19] McKee A. C. Daneshvar D. H. (2015). The Neuropathology of Traumatic Brain Injury. Handb. Clin. neurology 127, 45–66. 10.1016/b978-0-444-52892-6.00004-0 PMC4694720 25702209

[B20] McKerrow P. J. (1993). Echolocation - from Range to Outline Segments. Robotics Aut. Syst. 11, 205–211. 10.1016/0921-8890(93)90025-8

[B21] Memon Y. A. Motan I. Akbar M. A. Hameed S. Hasan M. U. (2016). Speech Recognition System for a Voice Controlled Robot with Real Time Obstacle Detection and Avoidance. Int. J. Electr. Electron. Data Commun. 4, 33–37.

[B22] Minetto M. A. Giannini A. McConnell R. Busso C. Torre G. Massazza G. (2020). Common Musculoskeletal Disorders in the Elderly: the Star Triad. Jcm 9, 1216. 10.3390/jcm9041216 PMC7231138 32340331

[B23] Mlinac M. E. Feng M. C. (2016). Assessment of Activities of Daily Living, Self-Care, and Independence. Arch. Clin. Neuropsychol. 31, 506–516. 10.1093/arclin/acw049 27475282

[B24] Nishimori M. Saitoh T. Konishi R. (2007). “Voice Controlled Intelligent Wheelchair,” in SICE Annual Conference 2007 (Takamatsu, Japan: IEEE), 336–340. 10.1109/sice.2007.4421003

[B25] Păsărică A. Bozomitu R. Cehan V. Lupu R. Rotariu C. (2015). “Pupil Detection Algorithms for Eye Tracking Applications,” in 2015 IEEE 21st International Symposium for Design and Technology in Electronic Packaging (SIITME) (Brasov, Romania: IEEE), 161–164. 10.1109/siitme.2015.7342317

[B26] Penaloza C. I. Nishio S. (2018). Bmi Control of a Third Arm for Multitasking. Sci. Robot. 3, eaat1228. 10.1126/scirobotics.aat1228 33141729

[B27] Peng D. G. Zhang H. Yang L. Li H. (2008). “Design and Realization of Modbus Protocol Based on Embedded Linux System,” in 2008 International Conference on Embedded Software and Systems Symposia (Chengdu, China: IEEE), 275–280. 10.1109/icess.symposia.2008.32

[B28] Penkert H. Baron J. C. Madaus K. Huber W. Berthele A. (2021). Assessment of a Novel, Smartglass-Based Control Device for Electrically Powered Wheelchairs. Disabil. Rehabilitation Assistive Technol. 16, 172–176. 10.1080/17483107.2019.1646817 31381862

[B29] [Dataset] Permobil (2022a). Compact Joystick. Available at: https://www.permobil.com/en-us/products/accessories/drive-controls/permobil-compact-joystick (Accessed Feb 25, 2022).

[B30] [Dataset] Permobil (2022b). Joystick Module w/Bluetooth. Available at: https://www.permobil.com/en-us/products/accessories/drive-controls/permobil-joystick-module-w-bluetooth (Accessed Feb 25, 2022).

[B31] [Dataset] Permobil (2022c). M3 Corpus. Available at: https://www.permobil.com/en-us/products/power-wheelchairs/permobil-m3-corpus (Accessed Feb 25, 2022).

[B32] Perrin S. Cassinelli A. Ishikawa M. (2004). “Gesture Recognition Using Laser-Based Tracking System,” in Sixth IEEE International Conference on Automatic Face and Gesture Recognition, 2004. Proceedings (Seoul, South Korea: IEEE), 541–546.

[B33] Puanhvuan D. Khemmachotikun S. Wechakarn P. Wijarn B. Wongsawat Y. (2017). Navigation-synchronized Multimodal Control Wheelchair from Brain to Alternative Assistive Technologies for Persons with Severe Disabilities. Cogn. Neurodyn 11, 117–134. 10.1007/s11571-017-9424-6 28348644 PMC5350091

[B34] Pulikottil T. B. Caimmi M. D’Angelo M. G. Biffi E. Pellegrinelli S. Tosatti L. M. (2018). “A Voice Control System for Assistive Robotic Arms: Preliminary Usability Tests on Patients,” in 2018 7th IEEE International Conference on Biomedical Robotics and Biomechatronics (Biorob) (Enschede, Netherlands: IEEE), 167–172. 10.1109/biorob.2018.8487200

[B35] Rabhi Y. Mrabet M. Fnaiech F. Gorce P. (2013). “Intelligent Joystick for Controlling Power Wheelchair Navigation,” in 3rd International Conference on Systems and Control (Algiers, Algeria: IEEE), 1020–1025. 10.1109/icosc.2013.6750981

[B36] Rabhi Y. Mrabet M. Fnaiech F. (2015). “Optimized Joystick Control Interface for Electric Powered Wheelchairs,” in 2015 16th International Conference on Sciences and Techniques of Automatic Control and Computer Engineering (STA) (Monastir, Tunisia: IEEE), 201–206. 10.1109/sta.2015.7505092

[B37] Ren Z. Yuan J. Meng J. Zhang Z. (2013). Robust Part-Based Hand Gesture Recognition Using Kinect Sensor. IEEE Trans. Multimed. 15, 1110–1120. 10.1109/tmm.2013.2246148

[B38] Rofer T. Mandel C. Laue T. (2009). “Controlling an Automated Wheelchair via Joystick/head-Joystick Supported by Smart Driving Assistance,” in 2009 IEEE International Conference on Rehabilitation Robotics (Kyoto, Japan: IEEE), 743–748. 10.1109/icorr.2009.5209506

[B39] Ruder S. (2016). An Overview of Gradient Descent Optimization Algorithms. arXiv Prepr. arXiv:1609.04747.

[B40] Rudigkeit N. Gebhard M. (2019). AMiCUS-A Head Motion-Based Interface for Control of an Assistive Robot. Sensors 19, 2836. 10.3390/s19122836 PMC6630260 31242706

[B41] Solea R. Margarit A. Cernega D. Serbencu A. (2019). “Head Movement Control of Powered Wheelchair,” in 2019 23rd International Conference on System Theory, Control and Computing (ICSTCC) (Sinaia, Romania: IEEE), 632–637. 10.1109/icstcc.2019.8885844

[B42] Sunny M. S. H. Zarif M. I. I. Rulik I. Sanjuan J. Rahman M. H. Ahamed S. I. (2021). Eye-gaze Control of a Wheelchair Mounted 6DOF Assistive Robot for Activities of Daily Living. J. NeuroEngineering Rehabil. 18, 173. 10.1186/s12984-021-00969-2 PMC8684692 34922590

[B43] Tangcharoensathien V. Witthayapipopsakul W. Viriyathorn S. Patcharanarumol W. (2018). Improving Access to Assistive Technologies: Challenges and Solutions in Low- and Middle-Income Countries. WHO South-East Asia J. Public Health 7, 84. 10.4103/2224-3151.239419 30136666

[B44] Taylor D. M. (2018). Americans with Disabilities: 2014. U. S. Census Bur. 2018, 1–32.

[B45] Thorp E. B. Abdollahi F. Chen D. Farshchiansadegh A. Lee M. H. Pedersen J. P. (2015). Upper Body-Based Power Wheelchair Control Interface for Individuals with Tetraplegia. IEEE Trans. Neural Syst. Rehabil. Eng. 24, 249–260. 10.1109/TNSRE.2015.2439240 26054071 PMC4742425

[B46] Toro-Hernández M. L. Kankipati P. Goldberg M. Contepomi S. Tsukimoto D. R. Bray N. (2019). Appropriate Assistive Technology for Developing Countries. Phys. Med. Rehabilitation Clin. N. Am. 30, 847–865. 10.1016/j.pmr.2019.07.008 31563175

[B47] Try P. Schöllmann S. Wöhle L. Gebhard M. (2021). Visual Sensor Fusion Based Autonomous Robotic System for Assistive Drinking. Sensors 21, 5419. 10.3390/s21165419 34450861 PMC8401834

[B48] Valk T. A. Mouton L. J. Otten E. Bongers R. M. (2019). Fixed Muscle Synergies and Their Potential to Improve the Intuitive Control of Myoelectric Assistive Technology for Upper Extremities. J. Neuroeng Rehabil. 16, 6–20. 10.1186/s12984-018-0469-5 30616663 PMC6323752

[B49] [Dataset] Wright C. (2022). PG Drives Technology. R-Net Technical Manual SK77981/14. Available at: Online http://sunrise.pgdrivestechnology.com/manuals/pgdt_rnet_manual_SK77981-14.pdf (Accessed Feb 25, 2022).

[B50] [Dataset] xArm Collaborative Robot (2022). xArm Collaborative Robot | UFACTORY (Accessed May 25, 2022).

[B51] Zhou S. Fei F. Zhang G. Mai J. D. Liu Y. Liou J. Y. (2013). 2d Human Gesture Tracking and Recognition by the Fusion of Mems Inertial and Vision Sensors. IEEE Sensors J. 14, 1160–1170.

